# Exploring Marine as a Rich Source of Bioactive Peptides: Challenges and Opportunities from Marine Pharmacology

**DOI:** 10.3390/md20030208

**Published:** 2022-03-13

**Authors:** Ishtiaq Ahmed, Muhammad Asgher, Farooq Sher, Syed Makhdoom Hussain, Nadia Nazish, Navneet Joshi, Ashutosh Sharma, Roberto Parra-Saldívar, Muhammad Bilal, Hafiz M. N. Iqbal

**Affiliations:** 1 Menzies Health Institute Queensland, School of Medical Science, Griffith University, Gold Coast Campus, Gold Coast, QLD 4222, Australia; ishtiaq.ahmed@griffithuni.edu.au; 2 Department of Regional Science Operations, La Trobe Rural Health School, Albury-Wodonga, Flora Hill, VIC 3690, Australia; 3 Department of Biochemistry, University of Agriculture Faisalabad, Faisalabad 38000, Punjab, Pakistan; mabajwapk@yahoo.com; 4 Department of Engineering, School of Science and Technology, Nottingham Trent University, Nottingham NG11 8NS, UK; farooq.Sher@ntu.ac.uk; 5 Fish Nutrition Lab, Department of Zoology, Government College University Faisalabad, Faisalabad 38000, Punjab, Pakistan; drmakhdoom90@gmail.com; 6 Department of Zoology, University of Sialkot, Sialkot 51040, Punjab, Pakistan; nadia.nazish@uskt.edu.pk; 7 Department of Biosciences, School of Liberal Arts and Sciences, Mody University of Science and Technology, Lakshmangarh, Sikar 332311, India; navybiotech@gmail.com; 8 Tecnologico de Monterrey, School of Engineering and Sciences, Centre of Bioengineering, Av. Epigmenio González No. 500, Fracc. San Pablo, Queretaro 76130, Mexico; asharma@tec.mx; 9 Tecnologico de Monterrey, School of Engineering and Sciences, Monterrey 64849, Mexico; r.parra@tec.mx; 10 School of Life Science and Food Engineering, Huaiyin Institute of Technology, Huai’an 223003, China; bilaluaf@hotmail.com

**Keywords:** marine biome, marine bioactive peptides, natural resources, bioactivities, pharmacology, biomedical, therapeutic attributes

## Abstract

This review highlights the underexplored potential and promises of marine bioactive peptides (MBPs) with unique structural, physicochemical, and biological activities to fight against the current and future human pathologies. A particular focus is given to the marine environment as a significant source to obtain or extract high-value MBPs from touched/untouched sources. For instance, marine microorganisms, including microalgae, bacteria, fungi, and marine polysaccharides, are considered prolific sources of amino acids at large, and peptides/polypeptides in particular, with fundamental structural sequence and functional entities of a carboxyl group, amine, hydrogen, and a variety of R groups. Thus, MBPs with tunable features, both structural and functional entities, along with bioactive traits of clinical and therapeutic value, are of ultimate interest to reinforce biomedical settings in the 21st century. On the other front, as the largest biome globally, the marine biome is the so-called “epitome of untouched or underexploited natural resources” and a considerable source with significant potentialities. Therefore, considering their biological and biomedical importance, researchers around the globe are redirecting and/or regaining their interests in valorizing the marine biome-based MBPs. This review focuses on the widespread bioactivities of MBPs, FDA-approved MBPs in the market, sustainable development goals (SDGs), and legislation to valorize marine biome to underlying the impact role of bioactive elements with the related pathways. Finally, a detailed overview of current challenges, conclusions, and future perspectives is also given to satisfy the stimulating demands of the pharmaceutical sector of the modern world.

## 1. Introduction

MBPs have gained significant research interests as robust alternatives to synthetic counterparts, becoming less effective with high drug-resistant issues. Therefore, naturally occurring sources with medicinal and pharmacological potentialities are ideal, owing to the ease in accessibility, free availability, natural abundance, being carbon neutral, and having fewer side effects than chemical-based synthetic formulations. Moreover, the growing technological advancement, scientific awareness, legislative authorities, and a broader variability of technological and analytical endorsement all support the exploitation of treasure of natural and underexploited sources from the marine biome [1,2]. In this context, the utilization of sustainable sources and the development of green technologies could be the key to going green. The value of “going green” has clearly transformed the pursuit of sustainable, recyclable, and socioeconomic friendlier products to fulfill the economic pressure by considering the high cost-effective ratio benefit. Therefore, the divergence from nonrenewable to renewable natural resources is fetching the center of interest in biomedical establishments. Technological legislation is a driving force and should be considered with care to develop green strategies for the cleaner obtainment of high-value biologically active products [1]. Several approaches are in practice to develop a state-of-the-art bio-based platform for various technological applications in bio- and non-bioindustries of the modern world. Green biotechnology has a noteworthy potential, of course in combination with 12 principles of green chemistry, to eradicate the generation of wasteful protection and deprotection steps [3]. The combination of green chemistry principles and modern biotechnology along with the employment of natural resources is urgently required to establish a sustainable future production and exploitation of the above-mentioned products.

MBPs are characteristically accessible from numerous natural sources, including marine biome, though in different extents with unique functionalities. Among several marine-derived compounds, MBPs are greener alternatives and have been broadly considered and utilized as pharmaceutically resourceful ingredients for numerous health determinations. Therefore, MBPs are being used to develop novel formulations in the biomedical, pharmaceutical, cosmeceutical, and nutraceutical industries. Owing to their advantageous features, such as robust affinity, functional reactivity, specificity, selectivity, etc., MBPs offer practical and positive replacements, compared with their synthetic counterpart formulations and chemical drugs [4,5]. Regardless of advantageous features, MBPs have been considered from diverse standpoints, i.e., based on (1) taxonomic sources, (2) biosynthesis pathways, (3) source of extraction, (4) ring and linear structures, (5) active fractions and compositional variations, (6) biologically active precursor molecules, etc. [2]. Irrespective of the category type, MBPs are protein-based specific fragments that solely depend on the activity based on the composition and amino acid sequence in that particular fragment [6]. However, the MBP activity indeed depends on the extraction and purification technique [2,7,8]. Thus far, an array of production, extraction, and purification approaches have been developed and exploited for MBPs [9,10,11]. From the above examples, an appropriately established methodology plays a significant role in the quality and quantity of the end-product of interests, specifically MBPs. It is also imperative to consider innumerable manipulating factors that can influence the complete performance of the entire isolation, extraction, and purification process and the overall product yield. For example, the source material’s physiochemical composition, matrix characteristics, material’s pretreatment considerations, solvent grade, type and concentration, reaction pH and temperature, pressure, reaction time, etc., are key influencing factors that should be considered before designing and running the experimental protocols [9]. The process efficacy of the entire extraction process and the end-product relies on (1) input parameters, (2) consideration of the nature of the substrate materials from the source, (3) interplay between the procedure and the substrate materials, and (4) chemistry of MBPs [2].

Considering the above critiques and opportunities herein, we sought to highlight the value, challenges, and opportunities that exist in MBPs with potent bioactivities and therapeutic potentialities. Following a brief introduction, a standardized initial methodological literature screening and inclusion/exclusion criteria were adopted to justify the scientific and literature theme. The next section concerns the marine biome and its potent MBPs with superior performance. The latter half of the paper addresses the generalized overview of bioactivities with biomedical and pharmaceutical potentialities to understand the fundamental impact and role of bioactive elements with related pathways. The FDA-approved MBPs in the market and MBPs with clinical trials, as well as sustainable development goals (SDGs) and legislation to valorize marine biome, are discussed. Finally, a detailed overview of current challenges, conclusions, and future perspectives is given to satisfy the stimulating demands of the pharmaceutical sector of the modern world.

## 2. Review of Methodological Approach: Inclusion/Exclusion Criteria

**Figure 1:**
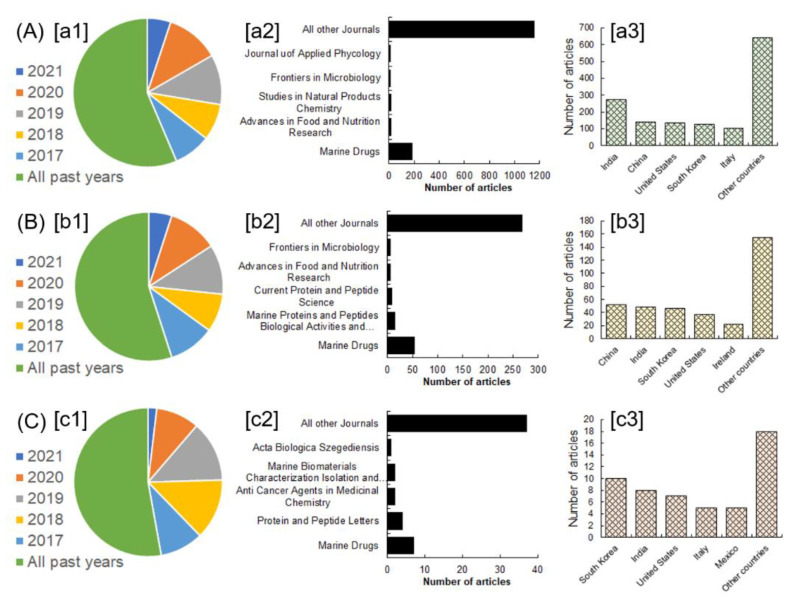
The literature screening results attained from the Scopus database. The letters A–C characterize the search terms: (**A**) bioactive compounds from marine sources; (**B**) bioactive peptides from marine sources; (**C**) biomedical applications of marine peptides. The letters [a1]–[c1] correspond to the number of articles from all years in that specific category of search term, [b2]–[c2] represents the number of articles published in different journals, and [a3]–[c3] represents the number of articles based on territory. Data were extracted from https://www.scopus.com, access on 25 April 2021.

The summarized review contents and given examples are well justified by using a standardized methodological approach that is based on screening the initial literature and inclusion/exclusion criteria. In this study, inclusion/exclusion criteria were also considered important to justify the most recent and relevant literature as per the conceptualized theme of the research. Following the initial screening step, the studies meticulously corresponding with the title theme were included in this discussion unless otherwise excluded to avoid the literature redundancy. Careful literature search queries were made in the Scopus and PubMed literature databases using any or many of the following terms: bioactive compounds from marine sources, bioactive peptides from marine sources, and biomedical applications of marine peptides. The active search filters were article title, abstract, and keywords. The literature screening results attained from the Scopus database are shown in Figure 1. The literature search queries were executed on 21 March 2021 and 25 April 2021 at Scopus, while for PubMed database search, the simultaneous search queries were performed on 21 March 2021 and 25 April 2021 at PubMed. Table 1 summarizes the literature screening results attained from the PubMed database.

## 3. Marine Biome—A Rich Source of MBPs

The marine biome represents the aquatic region and is considered the prime aquatic biome in the world with a plethora of untouched or underexploited sources of high-value interests, having around 500,000 to 10 million marine species [12]. Owing to the difficulty in reaching the lower/deeper zones of the marine biome, several MBPs have yet to be explored to identify and characterize marine biome; thus, marine biome constitutes a rich source of novel compounds with superior performance. As a major component of the biosphere, the aquatic biome covers almost 70% of the earth’s surface [13]. The aquatic biome is broadly divided into two central regions—namely, (1) freshwater regions and (2) marine regions. The freshwater regions of the aquatic biome comprise ponds/lakes, streams/rivers, and wetlands, while the marine region comprises neritic, oceanic, and benthic biomes. Other marine biome zones include intertidal, estuaries, and coral reefs. These alone have the highest biodiversity of all marine biomes. Considering all marine biomes, marine-originated species embrace about half of the total global biodiversity, with a considerable potential to extract products of interest. Thus, several marine biome sources have been broadly discovered and utilized to obtain novel bioactive compounds, e.g., MBPs. The aquatic organisms are classified into three basic categories: (1) plankton, including phytoplankton (bacteria and algae) and zooplankton (tiny animals that feed on phytoplankton); (2) nekton (invertebrates such as shrimp and vertebrates such as fish); (3) benthos (sponges, clams, sea stars, etc.).

Owing to this massive biodiversity, the marine biome is proved to be a highly advantageous and acceptable natural source that can fulfill the growing ecological and socioeconomic demands. Moreover, marine-based natural resources and/or their extracted bioproducts are easily accessible, have high bioactive efficacy, and exert no/fewer side effects [2], compared with the similar representative from synthetic formulations. In the marine biome, the complex habitat environment and the extreme living conditions, such as ultraviolet light exposure, variation in saltiness, thermal conditions of the environment, and limited/excessive nutrient availability, are the contributory aspects to produce MBPs. In consequence, the marine resources including microorganisms such as microalgae survive these extreme environments by rapidly acclimatizing to the new and surrounding atmosphere [14]. Such quick environmental adaptions further assist them to produce more stable and highly effective metabolites, which are biologically active and not common in other similar synthetic counterparts. Owing to their high protein content, microalgae and other marine sources are considered potential sources to produce both elementary proteins and therapeutic peptides [15]. Furthermore, seeing the enormous marine biome potential for industrial sections, the principle of “going green” has scrutinized this substitute search to eco-friendly, ecological, and sustainable materials with comparable socioeconomic benefits. The expansion of distinguishing practices or approaches to improve the cutting-edge platforms also supports the green agenda. The synergistic use of marine-derived natural resources, along with green and modern biotechnology, must be considered to unveil a sustainable production of high-value-added products with highly requisite features [2].

## 4. Marine Bioactive Peptides (MBPs)

**Figure 2:**
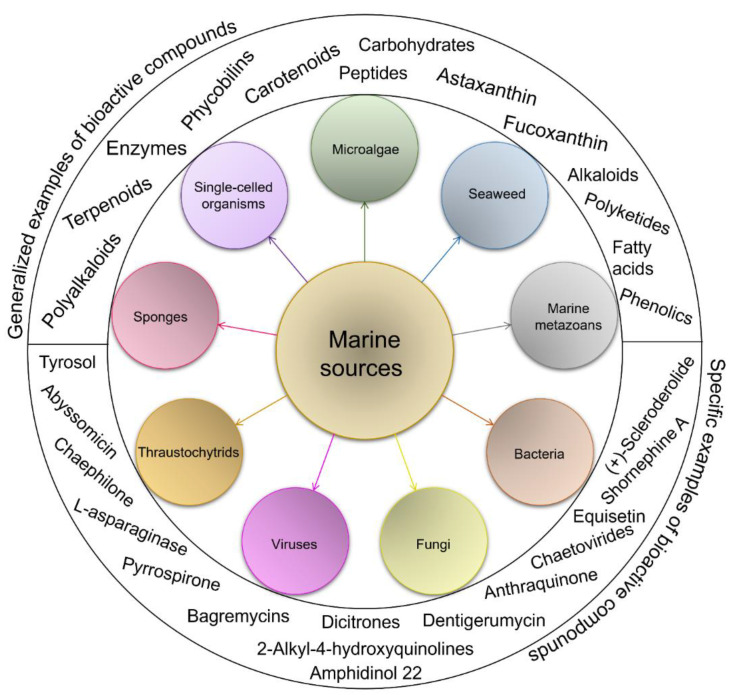
Various marine sources, along with their representative products or byproducts that have been either accepted or granted to enter the clinical trials.

The amino-acid-based organic substances joined by covalent bonds (e.g., amide or peptide bonds) are termed bioactive peptides (BPs). Based on the natural source, some peptides exist freely. However, the majority of the peptides are surrounded or encoded with respective parent protein molecules. In later cases, the pretreatments in the presence of related enzymes, such as proteolytic enzymes, facilitate the release of BPs in an efficient manner [2,16,17]. Such pretreatments cause the hydrolysis of the cell walls or cell membranes, as applied. Thus, the deployment of highly effective and suitable pretreatment supports the retrieval of intracellularly captured bioactive constituents, which are mainly not easy to extract via conventional extraction procedures [2]. Following enzyme facilitated pretreatment, once the encrypted peptides are released from their respective source materials, the amino acid composition and sequence govern the activity [17]. However, owing to the similar peptide length that ranges between 2 and 20 amino acids, most of the BPs share their structural and physicochemical features [18]. The broad bioactivity spectra of MBPs, such as antimicrobial, antiviral, anticancer, anticoagulant, antidiabetic, cardioprotective, immunomodulatory, neuroregenerative, appetite suppressing, etc. [19,20,21,22,23,24], have attracted the attention of biomedical, pharmaceutical, and nutraceutical sectors, with the hope that they can be used as a new frontier to diagnose, treat, or prevent various pathologies in human. Based on the evidence derived from the literature, some of the MBPs and/or their representative biologically active byproducts have gained considerable commercial values and seized the pharmaceutical market. From the perspective of the market status and share, ziconotide and brentuximab vedotin are two important representatives of MBPs and peptide derivatives, and both have successfully reached the market. The premier, Ziconotide (Prialt^®^), was obtained from a marine cone snail. It was the first peptide from the marine biome approved by the Food and Drug Administration (FDA) USA in 2004 to exploit and use against analgesics [25]. Later in 2011, FDA also approved another marine-derived drug, Adcetris^®^, to manipulate and use against cancer. Since then, numerous other MBPs have been evaluated for various phases of clinical related trials in the United States and Europe [13]. Figure 2 shows various marine sources along with their representative products or byproducts that have been either accepted or granted to enter the clinical trials. The commercial value of these therapeutic protein-based products was around USD 174.7 Billion in 2015. With a recent hike in interests, it is anticipated that this value will reach/cross USD 266.6 Billion in 2021 [26]. Several other marine-based peptides with different bioactivities and applications are summarized in Table 2.

## 5. Bioactivities—An Overview

As mentioned earlier, the marine biome having millions of macro- and microspecies (both animal and plants) is becoming highly significant [49,50]. This is also because the marine is considered a prolific source of structurally and functionally diverse MBPs at large, and MBPs in particular. The pharmacological activities, such as antimicrobial, antiviral, anticancer, anticoagulant, antidiabetic, cardioprotective, immunomodulatory, neuroregenerative, appetite suppressing, and many other MBPs, have been described in the literature [14,15,16,17,18,19,51,52]. Anticancer, anti-inflammatory, immunomodulating, antioxidant, hepatoprotective, and neuroprotective activities of phycobiliproteins from cyanobacteria and red algae are reported [15,53]. According to one estimate, the marine biome offers around 8000 species of red algae, which are highly enriched with several MBPs. An array of cyanobacterial-based MBPs, e.g., polypeptide or a hybrid of polyketide–polypeptide, are obtained via polyketide synthase and nonribosomal peptide synthase [54] and comprise exceptional characteristics, e.g., amino and/or hydroxy acids, heteroaromatic ring systems, and extended polyketide-derived units [55]. Likewise, several other BP-based functional entities, e.g., microcystin (a cyclic peptide), majusculamide 8 (depsipeptide), etc. have been isolated and/or extracted from marine-based blue-green algae [56,57]. Ghalamara et al. [58] proposed and extracted bioactive peptides-enriched and bioactive fractions from codfish blood, which shows notable antimicrobial properties and inhibits *Escherichia coli* growth [58].

Even though marine organisms at large, and microalgae, in particular, are highly enriched with diverse MBPs, their application as antimicrobials against human pathogens and diseases in aquaculture is still in its early stages [59]. With ever-rising heavy drug resistance and/or emergence/reemergence of new resistant microbial infections, there is a dire need to develop robust classes of natural antibiotics in combination with MBPs to treat the emerging/reemerging bacterial pathogens [60]. Diverse natural compounds with potent antimicrobial properties have been described in algae that belong to a wide range of chemical classes, including fatty acids, indoles, acetogenins, phenols, terpenes, and volatile, halogenated hydrocarbons [61]. Macro- and microalgae have grown to produce MBPs to pledge pathogenic bacteria [62], which are considered ubiquitous to their environment. Under the current scenario, effective treatment for a multi-drug-resistant *S. aureus* has become a challenge and extremely worrying concern [63]. Therefore, an alternative, novel antimicrobial agent is highly demanded.

**Figure 3:**
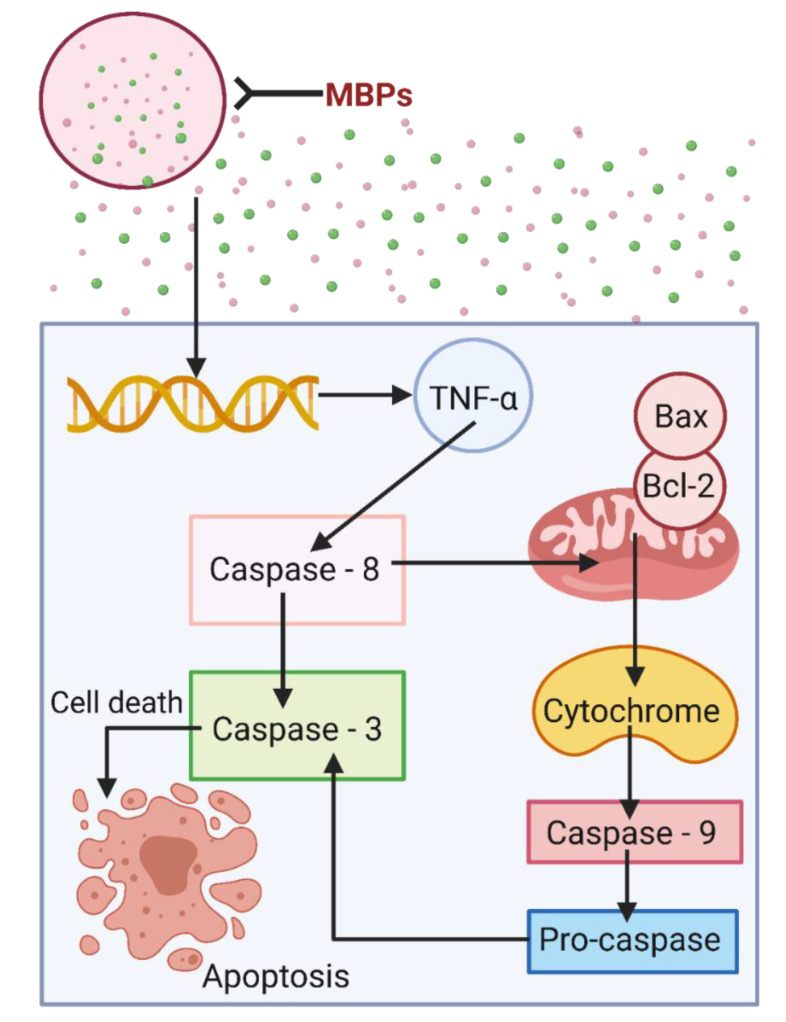
Anticancer potentialities of MBPs to abolish cancer cells and avoid metastasis via apoptosis mechanism. Created with BioRender.com and extracted under premium membership.

Cancer is a multifaceted disease in which abnormal cells divide overpoweringly and abolish normal body tissue. Owing to its molecular variations and consequential cellular effects, it has expanded extensive courtesy in terms of public health. Cancer involves several types that cause adversative consequences, e.g., leading to the growth of a cell mass with the capability to penetrate adjacent normal tissues and move to new locations via a mechanism known as metastasis (Figure S1) [64,65]. Figure S2 shows a detailed metastasis to new locations in (A) lungs and (B) brain shown in Figure S1. MBPs can attack the cancerous cells membrane by the processes called either necrosis or apoptosis that may cause cell death. In necrosis, the peptides target the negatively charged molecules on the cancer cell membrane and cause cell lysis, while in the case of apoptosis (Figure 3), they cause disruption of the mitochondrial membrane [66,67,68]. Amphipathic peptides have shown notable bioactivities and can easily be obtained from various marine-based natural resources [5,69]. Amphipathic peptides can be used to strengthen the natural defense against invasive pathogens. The prospective therapeutic application of MBPs has been established because of their wide-ranging bioactivity spectrum and the likelihood of not inducing resistance [70]. Initiation of extrinsic apoptotic activity pathways and inhibition of angiogenesis is an excellent example of killing action of MBPs. Owing to their capability to work synergistically, most of the MBPs, expressly antitumor peptides, are considered highly active in combination with the old style, but in practice, they are chemotherapeutic agents [5].

Based on the structural characteristics, they are mainly categorized into (1) cysteine-rich and β-sheet peptides, e.g., α- and β -defensins; (2) α-helices comprising peptides, e.g., LL-37 cathelicidin, cecropins, magainins, etc.; (3) peptide structures that are rich in glycine, proline, tryptophan, arginine, histidine, etc.; (4) peptides comprising a single disulfide bond, e.g., bactenecin. Further examples in the literature regarding some particular peptides having anticancerous activities are given in Table 2. Microalgal peptides with antiatherosclerotic activity have also been recognized, as described by Fan et al. [71]. Atherogenesis is an artery wall syndrome that encompasses several progressions, including cell adhesion, migration, differentiation, proliferation, and cell interaction with the extracellular matrix till the formation of atherosclerotic plaques, controlled by a complex network/cascade of cytokines and growth regulatory peptides. Besides the above-mentioned model examples, several other biological activities, such as anticoagulant, antidiabetic, cardioprotective, immunomodulatory, neuroregenerative, appetite suppressing, and others have fascinated researchers and strengthened the marine pharmacology sector [72,73,74]. Oxidative stress is a foremost reason for inflammatory events implicated in many of the above-mentioned diseases (e.g., neurodegenerative, cardiovascular, cancer, diabetes, etc.) [72,74].

## 6. FDA-Approved MBPs in the Market and Clinical Trials

Owing to the unique structural and multifunctional attributes, an array of MBPs have attained FDA-approved status, as already verified with numerous bioactivities, such as antimicrobials (antibacterial, and antifungal), antiviral, anticancer, antioxidant, antihypertensive, anticoagulant, antithrombotic, immunomodulatory, cholesterol-lowering activities, etc. The market-available MBP-based products have been developed using a unique combination of pristine counterparts and compositional alternation, as appropriate for medicinal, nutraceutical, and pharmaceutical uses. A plethora of BPs have been extracted from marine sources, treated, and purified, though using different materials and methods. However, a small fraction of those BPs have been legalized to move for clinical phase assessment, and even a few have accomplished to reach in the market. To avoid literature redundancy, selected FDA-approved MBPs are summarized in Table 3 with detailed information. In contrast, several other marine-derived biologically active compounds/products, including Pliditepsin, PM00104, Kahalalide F, Hemiasterlin, Spisulosine, Pseudopterosin A, Salinosporamide A, Tetrodotoxin, Conotoxin G, Bryostatin 1, and Plinabulin, are under clinical trials (phases I–III) [21,22,75,76,77].

## 7. Sustainable Development Goals (SDGs) and Legislation to Valorize Marine Biome

The sustainable development goals (SDGs) are blueprints that urge to attain better tomorrow and a more sustainable future. Moreover, a highly efficient transformation of innumerable marine resources into high-value entities, such as MBPs, as per SDGs, is of supreme interest. The potential of marine pharmacology pointedly subsidizes accomplishing 14 out of 17 of the United Nations (UN) SDGs (available online: https://sdgs.un.org/ -Last (accessed on 27 April 2021)). More specifically, with particular reference to SDG 14, “life below water” is a central theme for the sighting of biodiversity and the sustainable use of marine-based natural resources. Promotion of resource efficiency and technological development contribute to SDG 12 (Responsible Consumption and Production) and SDG 9 (Industry, Innovation, and Infrastructure). Therefore, in order to achieve SDG 3, with good health and well-being theme, the well-developed and effectively deployed policy frameworks and regulations or a combination of SDG measures are much needed, including the expansion of new products in the medical and pharmaceutical industries. The establishment of partnerships between governments, industry, civil society, and the scientific sector contributes to SDG 17 (Partnerships for the Goals). As confirmed most recently by Agenda 2030, the ecosystem-based approach is vital to “conserve and sustainably use the oceans, seas and marine resources for sustainable development” (SDG 14). At the sea-basin level, a close regional alliance of Member States within pertinent regional sea conventions helps synchronize the regional execution and valuation of ocean-related SDGs.

European Union (EU) legislation supports marine spatial planning (MSP) to balance the maritime economy while protecting and valorizing biodiversity. Several complementary policies have been regulated, i.e., the regulation of fisheries through the Common Fisheries Policy (CFP), EU Biodiversity Strategy to 2020, EU Regulation 1143/2014 on Invasive Alien Species, and the control of the input of nutrients and chemicals into waters through the Water Framework Directive (WFD), etc. The EU Marine Strategy Framework Directive, the environmental pillar of the EU maritime policy, introduced the principle of ecosystem-based marine spatial planning and provided a supportive framework for national initiatives toward spatial planning, designed for achieving good status for the environment. In summary, successful execution of the Marine Directive will be dynamic if the Integrated Maritime Policy is to be delivered as intended. To provide further insight on the current policy frameworks and regulations, some of the EU directives and legislation are summarized in Table S1.

## 8. Conclusions, Current Challenges, and Future Considerations

In summary, marine biotechnology and pharmacology have infinite potential to formulate/synthesize new MBPs that show a dynamic role in bio- and non-bioindustries of the modern world. MBPs obtained by following green chemistry agenda principles using naturally existing sources from marine biomes such as microalgae have succeeding merits, including natural abundance, ease in availability throughout the year, materials renewability and sustainability, carbon-neutral aspects, re-processibility with a zero-waste SDG agenda, facile synthesis options with possibilities to scale up, net positive and high cost-effective ratio, no or minimal consumption of harsh chemicals/reagents, and no or less toxic contaminants/byproducts, etc. Nonetheless, colossal steps have already been reserved and taken in the past years; however, focused and genetically positioned research is necessary to further strengthen the marine pharmacology consideration for a better tomorrow.

Regardless of ever-growing scientific awareness and technological advancement, several challenges still exist to address valorize MBPs from marine origins. These include (1) reachability ease to the unexplored biodiversity, (2) standardized procedural isolation (regardless of the source variability), (3) cost-effective handling of the extracted products, (4) stability maintenance under standardized environment for any or many products at the same time (regardless of the compositional variability), (5) possible scale-up probability, (6) sustainable marketing and commercialization, all of which are vital challenges and should be considered with care. A considerable amount of information is available in the literature about numerous potential aspects of marine biotechnology and pharmacology. However, substantial critiques are still unresolved, which necessitate future studies. Despite contemporary scientific advancements in marine biotechnology, extensive research with verified employability of marine sources is needed in this line of research.

## Figures and Tables

**Table 1:** The literature screening results attained from the PubMed database. Data were extracted from https://pubmed.ncbi.nlm.nih.gov/, access on 25 April 2021.

**Search Terms**	**Total Articles**	**Total Number of Articles Published and Filtered with Best Match Term on**					
		**2021**	**2020**	**2019**	**2018**	**2017**	**All Past Years**
Bioactive compounds from marine sources	995	74	172	147	97	101	404
Bioactive peptides from marine sources	307	15	41	52	36	34	129
Biomedical applications of marine peptides	76	3	10	15	11	6	31

**Table 2:** Marine-based peptides (MBPs) with different bioactivities and applications.

Peptide Name	Structure	Chemical Formula	Molar Mass (g/mol)	Class or Type of Chemical Compounds	Bioactivities and/or Proposed Applications	References
Astaxanthin	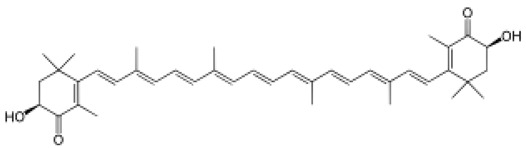	C_40_H_52_O_4_	596.84	Terpenes	Nutraceutical and pharmaceutical applications	Messina et al. [27]
Cyclopolypeptide, e.g., α-amanitin, rolloamide A	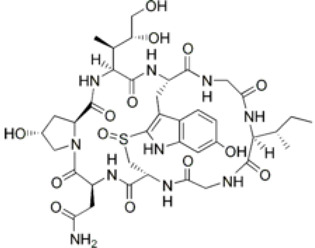	C_39_H_54_N_10_O_14_S	918.97	Cyclic octopeptide	Antibacterial activity, antifungal activity, anthelmintic activity	Dahiya et al. [28]
Plitidepsin	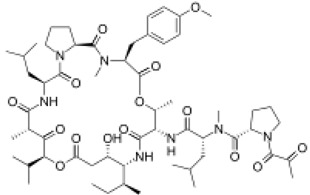	C_57_H_87_N_7_O_15_	1110.357	Cyclic depsipeptide	Effective against various cancers, e.g., breast, thyroid, lung, etc.	Leisch et al. [29]
Borophycin	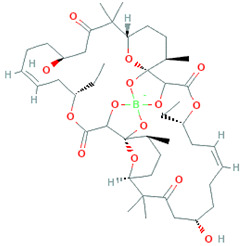	C_44_H_68_BO_14_	831.8	Organic compounds	Various carcinoma types, e.g., epidermoid and human colorectal adenocarcinoma	Nowruzi et al. [30]
Cryptophycin-52	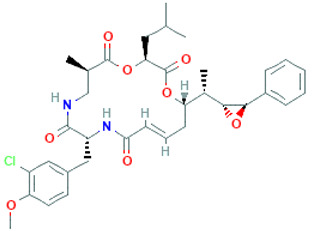	C_35_H_43_ClN_2_O_8_	655.2	Depsipeptide	Tumor cell lines	Nowruzi et al. [31]
Fucoxanthin	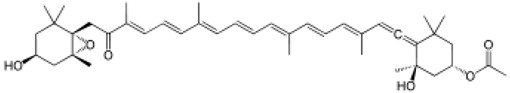	C_42_H_58_O_6_	658.920	Carotenoids	Gastric cancer	Zhu et al. [32]
Pardaxin	_	C_154_H_248_N_36_O_45_	3323.8	Cationic peptide	Oral squamous cell carcinoma	Han et al. [33]
Hepcidin	_	C_113_H_170_N_34_O_31_S_9_	2789.4	Cationic amphipathic peptide	Human cervical carcinoma, hepatocellular carcinoma, breast adenocarcinoma cell line	Hassana et al. [34]; Chang et al. [35]; Chen et al. [36]
Hemiasterlin	_	C_30_H_46_N_4_O_4_	526.7	Peptide	Inhibitory effect on microtubule assembly, cell cycle arrest, Apoptosis induction	Lai et al. [37]
Aurilide	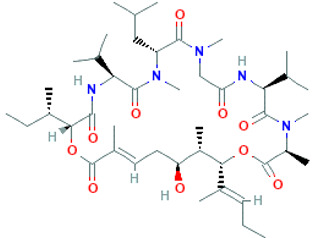	C_44_H_75_N_5_O_10_	834.1	Depsipeptides	Human lung tumor, leukemia, renal, and prostate cancer cell lines	Sato et al. [38]; Han et al. [39]; Suenaga et al. [40]
Desmethoxymajusculamide C	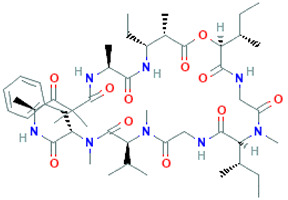	C_49_H_78_N_8_O_11_	955.2	Cyclic depsipeptide	Human colon HCT-116	Simmons et al. [41]
Diazonamide A	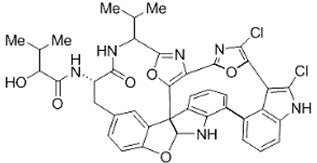	C_40_H_34_CI_2_N_6_O_6_	765.6	Oxazoles	Human tumor cells	Lachia and Moody [42]
PG155	_	_	15,500	Polypeptide	Potent antiangiogenic activity	Zheng et al. [43]
C-phycocyanin (e.g., Phycocyanobilin)	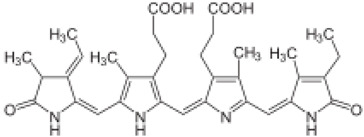	C_33_H_38_N_4_O_6_	586.7	Phycobiliprotein	Apoptosis induction	Li et al. [44]
Kahalalide F	_	C_75_H_124_N_14_O_16_	1477.9	Depsipeptide	Ovaries, breast, prostate, colon, and liver tumor cells	Sewell et al. [45]
Vitilevuamide	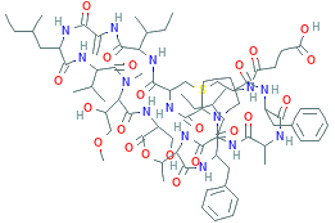	C_77_H_114_N_14_O_21_S	1603.9	Cyclic peptide	Lymphocytic inhibition of tubulin polymerization	Edler et al. [46]
Tachyplesin	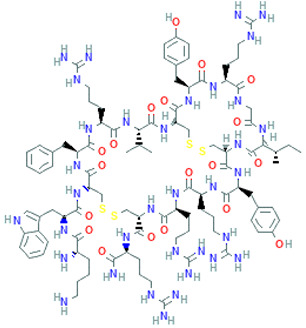	C_99_H_151_N_35_O_19_S_4_	2263.8	Cationic Peptides	Prostate, Melanoma, and endothelial cancer cell	Chen et al. [47]
Thiocoraline	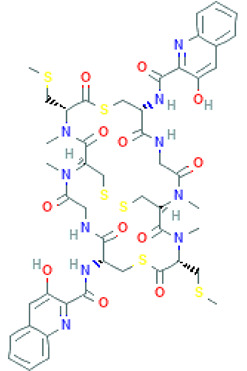	C_48_H_56_N_10_O_12_S_6_	1157.4	Depsipeptides	Human colon cancer	Erba et al. [48]

**Table 3:** Selected FDA-approved MBPs.

Compound Name	Formula	Molar Mass	CAS Number	Source	Natural/Derivative	Legal Status	Bioavailability ^1^	Elimination Half-Life	Applications
Ziconotide (intrathecal ziconotide)	C_102_H_172_N_36_O_32_S_7_	2639.14 (g/mol)	107452-89-1	Cone Snail	Natural product	Prescription only	50%	2.9 to 6.5 h	Analgesics
Adcetris (Brentuximab vedotin)	C_6476_H_9930_N_1690_O_2030_S_40_	149.2–151.8 (kg/mol)	914088-09-8	*Dolabella auricularia*	Derivative	Prescription only	50–80%	Approximately 4 to 6 days	Cancer treatment, treatment for patients with cutaneous T-cell lymphoma
Dactinomycin	C_62_H_86_N_12_O_16_	1255.438 (g/mol)	50-76-0	*Streptomyces parvullus*	Derivative	Prescription only	Not Available	36 h	Cancer treatment including for Gestational trophoblastic neoplasia, Wilms’ tumor, Rhabdomyosarcoma, Ewing’s sarcoma
Bacitracin (Baciim)	C_66_H_103_N_17_O_16_S	1422.71 (g/mol)	1405-87-4	*Bacillus subtilis*	Natural product	Prescription-only for injection and OTC	Not Available	Not Available	Acute and chronic localized skin infections
Dutasteride (Avodart)	C_27_H_30_F_6_N_2_O_2_	528.539 (g/mol)	164656-23-9	_	Synthetic	Prescription-only	60%	4–5 Weeks	Treat enlarged prostate, Prostate cancer, hormone therapy
Curacin A	C_23_H_35_NOS	373.60 (g/mol)	155233-30-0	*Lyngbya majuscula*	Natural product	Not Available	Not Available	Not Available	Cancer treatment
Eribulin (Halaven)	C_40_H_59_NO_11_	729.908 (g/mol)	253128-41-5	Marine Sponge	_	Prescription-only	Not Available	40 h	Cancer treatment
Trabectedin (Yondelis)	C_39_H_43_N_3_O_11_S	761.84 (g/mol)	114899-77-3	Marine Tunicate		Prescription-only	Not Available	180 h	Antitumor chemotherapy medication for the treatment of advanced soft-tissue sarcoma and ovarian cancer

^1^: In pharmacology, bioavailability refers to absorption and is the fraction (%) of an administered drug that reaches the systemic circulation.

## Data Availability

Not applicable.

## Supplementary Materials

The following are available online at https://www.mdpi.com/article/10.3390/md20030208/s1, Figure S1: Schematic representation of cancer metastasis to new locations. Cancer involves several types that cause adversative consequences, e.g., leading to the growth of a cell mass with the capability to penetrate and move to adjacent normal tissues. In metastasis, cancer cells break away from where they first formed (primary cancer site), travel through the blood stream, and form new tumors (metastatic tumors) in other parts of the body (secondary cancer site). Created with BioRender.com and extracted under premium membership. Figure S2: A detailed metastasis to new locations (**A**) lungs and (**B**) brain, with reference to the model locations marked in Figure S1. Created with BioRender.com and extracted under premium membership. Table S1: EU directives and legislation that support current policy frameworks and regulations in the field of marine environmental policy.
