# Supplementary material for: Exploring Marine as a Rich Source of Bioactive Peptides: Challenges and Opportunities from Marine Pharmacology
Source: Mar Drugs. 2022 Mar 13;20(3):208. doi: 10.3390/md20030208 (PMC8948685; doi:10.3390/md20030208)
Supplement: Supplementary file 1 [file marinedrugs-20-00208-s001.zip › marinedrugs-1613914-supplementary.pdf]

Supplementary material

Exploring Marine as A Rich Source of Bioactive Peptides: Challenges and Opportunities from Marine Pharmacology

Ishtiaq Ahmed [1], Muhammad Asgher [2], Farooq Sher [3], Syed Makhdoom Hussain [4], Nadia Nazish [5], Navneet Joshi [6], Ashutosh Sharma [7], Roberto Parra-Saldívar [8], Muhammad Bilal [9], and Hafiz M.N. Iqbal [8,*]

[1]Gold Coast Campus, Menzies Health Institute Queensland, School of Medical Science, Griffith University, Gold Coast, QLD 4222, Australia; ishtiaq.ahmed@griffithuni.edu.au (I.A.).

[2] Department of Biochemistry, University of Agriculture Faisalabad, Pakistan; mabajwapk@yahoo.com (M.A.).

[3] Department of Engineering, School of Science and Technology, Nottingham Trent University, Nottingham NG11 8NS, UK; Farooq.Sher@ntu.ac.uk (F.S.).

[4] Fish Nutrition Lab, Department of Zoology, Government College University, Faisalabad, Pakistan; drmakhdoom90@gmail.com (S.M.H.).

[5] Department of Zoology, University of Sialkot, Pakistan; nadia.nazish@uskt.edu.pk (N.N.).

[6] Department of Biosciences, School of Liberal Arts and Sciences, Mody University of Science and Technology, Lakshmangarh, Sikar 332311, Rajasthan, India; navybiotech@gmail.com (N.J.).

[7] Tecnologico de Monterrey, School of Engineering and Sciences, Centre of Bioengineering, Av. Epigmenio González No. 500, Fracc. San Pablo 76130, Queretaro, Qro, Mexico; asharma@tec.mx (A.S.).

[8] Tecnologico de Monterrey, School of Engineering and Sciences, Monterrey 64849, Mexico; r.parra@tec.mx (R.P.S.); hafiz.iqbal@tec.mx (H.M.N.I.).

[9] School of Life Science and Food Engineering, Huaiyin Institute of Technology, Huai'an 223003, China; bilaluaf@hotmail.com (M.B.).

* Correspondence: hafiz.iqbal@tec.mx (H.M.N.I.)

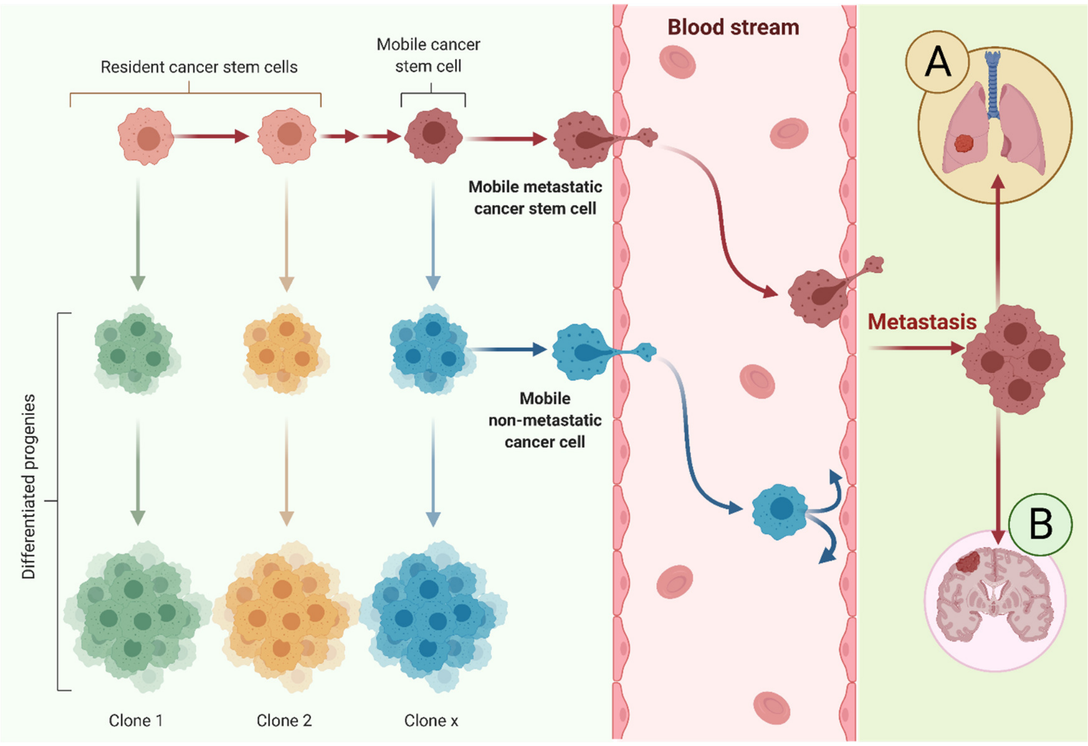

Figure S1. Schematic representation of cancer metastasis to new locations. Cancer involves several types that cause adversative consequences, e.g., leading to the growth of a cell mass with the capability to penetrate and move to adjacent normal tissues. In metastasis, cancer cells break away from where they first formed (primary cancer site), travel through the bloodstream, and form new tumors (metastatic tumors) in other parts of the body (secondary cancer site). *Created with BioRender.com and extracted under premium membership.*

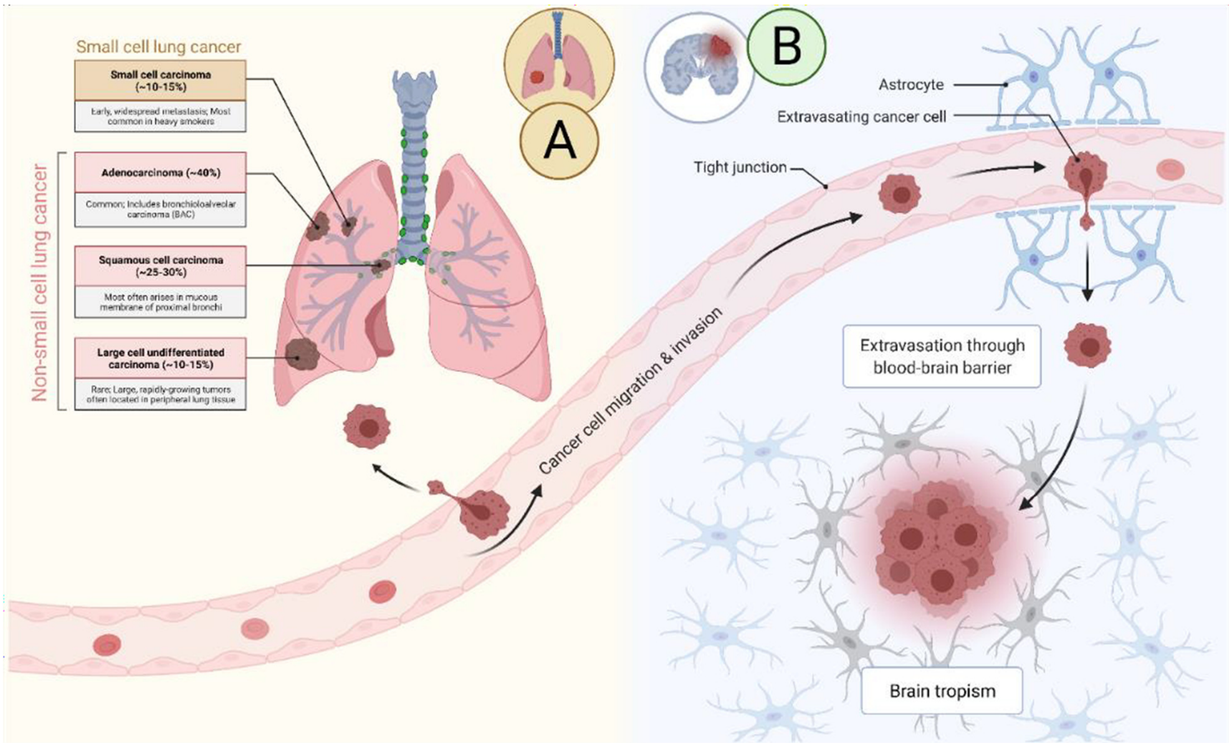

Figure S2. A detailed metastasis to new locations of (**A**) lungs and (**B**) brain, with reference to the model locations marked in Figure S1. *Created with BioRender.com and extracted under premium membership.*

Table S1. EU directives and legislation that support current policy frameworks and regulations in the field of marine environmental policy.

**Policy**	**Remarks**
Directive 2008/56/EC	A framework for community action in the field of marine environmental policy (Marine Strategy Framework Directive).
Directive 2006/113/EC	Directive on the quality required for shellfish waters.
Directive 91/692/EEC	Standardizing and rationalizing reports on the implementation of specific Directives relating to the environment.
Directive 2001/42/EC	Assessment of the effects of specific plans and programs on the environment requires a formal environmental evaluation of specific plans and programs (e.g., marine spatial plans), including those relating to fisheries and aquaculture.
Directive 2000/60/EC	A framework for Community action in the field of water policy.
Directive 2008/56/EC	Implementation of the Marine Strategy Framework Directive.<br>The Marine Strategy Framework Directive aims to achieve Good Environmental Status (GES) of the EU's marine waters by 2020 and to protect the resource base upon which marine-related economic and social activities depend. It is the first EU legislative instrument related to the protection of marine biodiversity, as it contains the explicit regulatory objective that "biodiversity is maintained by 2020".
Marine Strategy Framework Directive (MSFD) 2020	A better understanding of the pressures and impacts of human activities on the sea, and their implications for marine biodiversity, their habitats, and the ecosystems they sustain.
